# A Verification of Cicuta Viroso

**Published:** 1888-04

**Authors:** Alfred Heath

**Affiliations:** London


### A VERIFICATION OF CICUTA VIROSA.

ALFRED HEATH, ESQ., LONDON.

M. L., a domestic servant of mine, aged twenty-four, came to her mistress about eight P. M., asking for brandy. Before anything could be done she suddenly fell on the floor in a violent epileptic fit, in which she remained until two A. M., six hours. The following were her symptoms: Eyes staring, unaffected by light, total insensibility, at one time starting up and striking forward both fists at once at some imaginary person, then throwing her arms violently to the right and left, striking heavily the sofa on which she lay; before, at times, it could be prevented, clenching the teeth and biting the tongue, clenching the hands, and various other movements of a clonic character. Then all at once the symptoms would change into the most rigid tonic spasms —limbs and body set as if made of steel and immovable. I knew nothing of the cause of this, and the girl has been in apparent health. I gave her some of the most usual homœopathic remedies, but without any effect whatever. Suddenly she began a different movement, a violent *trembling and jerking of the right arm.* This made me think of Cicuta, although in the pathogenesis it chiefly affects the left arm; but as many of her other symptoms were covered by Cicuta, I immediately got the first bottle within reach, 3C, and poured two or three drops between her teeth. In less than ten minutes she looked at me, perfectly conscious, and asked me what was the matter. This occurred more than two years since, and she has never had the slightest return of the trouble up to this time.

